# Estrogen Receptors and Enzymes Involved in Estrogen Synthesis as Breast Cancer Treatment Targets

**DOI:** 10.3390/ijms27156823

**Published:** 2026-07-30

**Authors:** Barbara Licznerska, Hanna Szaefer, Hanna Sobierajska, Wanda Baer-Dubowska

**Affiliations:** Poznan University of Medical Sciences, Department of Pharmaceutical Biochemistry, 60-806 Poznań, Poland; barlicz@ump.edu.pl (B.L.); hszaefer@ump.edu.pl (H.S.); bernatha@ump.edu.pl (H.S.)

**Keywords:** breast cancer, estrogen receptors, aromatase, steroid sulfatase, 17β-hydroxysteroid dehydrogenases

## Abstract

Estrogens, particularly the most active 17β-estradiol (E2), play a crucial role in the initiation and development of breast cancer. Therefore, current treatment strategies are based on interfering with estrogen receptors (ERs) via pure antiestrogens or selective modulators (SERMs) or by inhibiting estrogen synthesis through key enzymes in this pathway. While SERMs are generally effective, resistance often develops. Moreover, they cannot be used to treat triple-negative breast cancers (TNBCs). Three critical enzymes in the estrogen synthesis pathway, namely aromatase, sulfatase, and 17β-hydroxysteroid dehydrogenases, are of particular interest as drug targets. Some, such as aromatase inhibitors, are already used in clinical practice, while the other two remain under intensive investigation. Several comprehensive reviews have been published on ER modulators or individual enzyme inhibitors. This paper attempts to summarize recent data on modulators/inhibitors of both the ER and the major enzymes involved in estrogen biosynthesis and offers a perspective on their further development.

## 1. Introduction

Approximately 70% of breast cancers are estrogen-dependent. Therefore, targeting either the action or the formation of estrogens is the major approach to treat these cancers. To block the action of estrogens, several estrogen receptor (ER) inhibitors, mainly ERα, have been developed, including pure antiestrogens and selective estrogen receptor modulators (SERMs). While the latter are generally effective for both breast cancer treatment and prophylaxis, some patients develop resistance [1]. Several factors may contribute to this process, and breast tumor heterogeneity and microenvironment are probably the most important [2].

Moreover, triple-negative breast cancer (TNBC), which accounts for approximately 15% of total breast cancer cases, does not respond to ER modulators due to the absence of ERα. Possible targets for improving TNBC include estrogen signaling through membranous ERα-36, which could reactivate silenced ERα. Inhibition of enzymes involved in estrogen synthesis might be applicable, probably not only to the treatment of ERα+ breast cancer.

Like other primates, humans can synthesize sex steroid hormones independently of gonadal sources by secreting dehydroepiandrosterone (DHEA) and its sulfated derivative, dehydroepiandrosterone sulfate (DHEA-S), which are then locally metabolized into active hormones [3]. Therefore, in postmenopausal women, sex steroids, including estrogens, are predominantly produced in extragonadal tissues from adrenal precursors, a system termed “intracrinology” [4]. Three enzymes are particularly important in estrogen synthesis from DHEA and DHEA-S, both in gonadal and intracrine contexts: aromatase (CYP19), steroid sulfatase (STS), and 17β-hydroxysteroid dehydrogenases (HSD17B). Beyond their primary activities, these enzymes contribute to the inactivation of steroid receptor ligands, facilitating controlled specific substrate fluxes and finely tuned physiological regulation. Therefore, they are of particular interest as druggable targets. While aromatase inhibitors are already used in clinical practice for the treatment of breast cancer, only one sulfatase inhibitor has reached clinical trials. HSD17B inhibitors, until the early 2000s, raised relatively little interest. However, subsequent years have brought a large number of potential modulators of these enzymes [5]. The HSD17B family comprises several distinct types. HSD17B1, the most studied member thus far, catalyzes the reduction of the weakly estrogenic estrone (E1) to the most potent estrogen, 17β-estradiol (E2), which plays a major role in the etiology of breast cancer. HSD17B1 also facilitates the conversion of DHEA into 5-androstene-3β,17β-diol, a steroid hormone whose levels rise after menopause. The regulation of the interplay between HSD17B1 and HSD17B2, which catalyze opposite reactions, is of particular interest in a therapeutic context. However, this concept still lacks a comprehensive preclinical and clinical evaluation. In addition, in searching for the new modulators or inhibitors of specific estrogen synthesis enzymes, various groups developed molecules that possess both ER modulating and key estrogen biosynthesis pathways inhibition properties [6,7,8]. However, most of these molecules were not tested in patients.

This review presents the current understanding of ER and major enzymes involved in estrogens’ synthesis, highlights their potential as drug targets, and explores future development prospects.

## 2. Estrogen Receptors and Their Modulation

### 2.1. Estrogen Receptors and Signaling Mechanisms

Estrogen receptors (ERs) are members of the superfamily of ligand-activated transcription factors that translate hormonal signals into a wide range of physiological responses across multiple organs, including the normal growth, proliferation, and differentiation of breast tissue [9]. Two major closely related estrogen receptor subtypes, ERα and ERβ, are encoded by separate genes and show distinct expression patterns across tissues [10,11,12]. In addition, a new type of estrogen-binding protein—the G Protein-Coupled Estrogen Receptor (GPER1)—was discovered [13,14,15]. While ERα and ERβ are mostly expressed in reproductive tissues, ERβ is also found in non-reproductive organs (e.g., lung, adrenal glands, adipose tissue, and brain). Both ER variants are expressed at low levels in normal breast tissue; however, ERα is expressed at higher levels than ERβ in breast cancer cells [12]. Moreover, in contrast to ERα, activation of ERβ is generally associated with inhibition of proliferation and induction of apoptosis. These effects, however, vary by tissue, cellular environment, transcriptional coactivators, and ERα co-expression [16].

ERs play a key role in the activation of signaling pathways controlled by estrogens. Binding of E2 to ER leads to the formation of complexes that can bind directly or indirectly to DNA, initiating the signaling events ultimately regulating gene expression. Therefore, E2-mediated signaling mechanisms are divided into genomic and non-genomic. Moreover, genomic signaling can be further divided into direct and indirect signaling [17].

In direct genomic signaling, ERα and ERβ act as ligand-activated transcription factors. Upon binding E2 to ERα or ERβ in the cytoplasm, a conformational change occurs, inducing receptor dimerization. The complex is then translocated to the nucleus, where it binds to E2 response elements (EREs). While EREs have been identified in several gene promoters and regulatory regions, it has been reported that approximately one-third of human genes regulated by ER do not contain ERE sequence elements [18,19]. In this case, ER complexes act indirectly through protein–protein interactions with the other transcription factors and response elements, such as activator protein-1 (AP-1) and specificity protein-1 (SP-1). ERα can also interact with the c-rel subunit of the nuclear factor-κB (NF-κB) complex, preventing NF-κB from binding to cytokine gene promoters, and the nuclear ATF-1/cAMP response element binding protein [20,21].

In non-genomic E2-mediated signaling activation, the relatively recently discovered ER, namely GPER1, is involved. This rapid way of signaling activation often leads to the subsequent production of intracellular second messengers, cAMP regulation, and protein-kinase activation of signaling cascades that result in indirect changes in gene expression [22,23,24]. GPER1, bound to estrogens, promotes estrogen-dependent activation of adenylyl cyclase and epidermal growth factor receptor (EGFR). Phosphorylation of different transcription factors by the kinases mentioned previously can change their function and ability to bind to genomic regions, resulting in altered gene expression patterns. Among the transcription factors affected by these signaling mechanisms are CREB, CCAAT-enhancer-binding protein beta (C/EBPβ), the NF-κB complex, and the signal transducer and activator of transcription (STAT) family [25,26,27,28,29].

Besides the membrane-bound estrogen receptor GPER1, some variants of the ERα and ERβ have been associated with non-genomic estrogen signaling [13,14]. It has been suggested that non-genomic actions of ERα and ERβ could be mediated by a subpopulation of receptors located at the cell membrane that can activate intracellular signaling cascades [30]. Among ERα splicing variants related to non-genomic estrogen signaling, short forms of the full-length ERα, ERα-46 (46-kDa), or ERα-36, are often mentioned [24,31].

Between the genomic and non-genomic signaling activation mechanisms, specific “cross-talk” has been described, which involves protein–protein interactions of components of both pathways [32]. Thus, the combination of the two classical estrogen receptor regulation pathways can result in enhanced transcriptional activity in specific tissues and physiological processes. Dysfunction of any element of these pathways, initiated by binding E2 to ER, can contribute to breast cancer development. Therefore, modulation of ERs is, so far, the first-line breast cancer treatment and a prophylactic target.

### 2.2. Selective Modulators and Degraders of Estrogen Receptors

Selective estrogen receptor modulators (SERMs) are a varied group of nonsteroidal compounds that act as either agonists or antagonists of estrogen receptors in a target gene- and tissue-specific manner. Tamoxifen, the first SERM described, was discovered accidentally after being synthesized in the 1960s in search of new contraceptive agents. It has now been used for over 50 years in breast cancer therapy and as one of the first synthetic chemopreventive agents. Unlike E2, antagonistic molecules such as tamoxifen induce an inactive conformation of ERα, recruiting transcriptional corepressors [33]. The tissue selectivity of TAM means that although it acts as an antiestrogen in the breast, it may have weak estrogen-like agonist effects in other tissues, such as bone or endometrium. In addition to tamoxifen, other SERMs (raloxifene, ospemifene, lasofoxifene, and bazedoxifene) are commonly used. Due to differences in pharmacokinetics and affinity for the ER, these drugs have different clinical uses, including breast cancer treatment and prevention, vasomotor and genitourinary syndromes of menopause, and prevention and treatment of osteoporosis [34]. Unfortunately, although SERMs are usually relatively effective, they are associated with serious side effects over time, and, more importantly, resistance to SERM treatment is very common, driven by complex, difficult-to-bypass mechanisms.

ERs can not only be blocked, as with SERMs, but also degraded, thereby inhibiting estrogen-induced signaling pathways. Such degrading compounds include selective estrogen receptor degraders (SERDs), pure ER antagonists that may overcome resistance to endocrine therapy in breast cancer. Fulvestrant, a 7α-alkylsulfinyl analog of E2, was the first SERD developed. SERDs bind to ERs, preventing their nuclear translocation or binding to EREs, ultimately leading to their proteasomal degradation. Regarding fulvestrant, its bioavailability is limited even when administered intramuscularly [35]. Currently, several oral SERDs are in clinical trials (e.g., elacestrant, camizestrant, giredestrant, imlunestrant, and amcenestrant). Moreover, new clinical trials are underway to evaluate complete estrogen receptor antagonists (CERANs), proteolysis-targeting chimeras (PROTACs), and selective estrogen receptor covalent antagonists (SERCAs) [36]. None of these studies have been completed.

### 2.3. The Attempts of ER Reactivation in Triple-Negative Breast Cancer and New Concepts of Treatment

Triple-negative breast cancer (TNBC) is a highly aggressive subtype of breast cancer, defined by the absence of ER and progesterone receptor (PR), as well as the lack of human epidermal growth factor receptor 2 (HER2) overexpression. Therefore, estrogen-related targeted therapy cannot be applied in this type of breast cancer. Thus, TNBCs are usually treated with conventional non-targeted chemotherapy using anthracyclines, platinum agents, taxanes, or antimetabolites. The discovery of the tumor microenvironment (TME) and immune cell crosstalk in TNBC opened up new therapeutic options [37]. Immunotherapy has significantly improved outcomes in advanced TNBC, with atezolizumab or pembrolizumab combined with chemotherapy representing the standard first-line approach for inoperable, locally advanced, or metastatic TNBC. For patients with germline *BRCA1/2* mutations and HER2-negative tumors, PARP inhibitors such as olaparib and talazoparib are recommended [38,39]. Unfortunately, despite therapeutic advances, TNBC remains associated with high relapse rates and poor long-term outcomes.

Although immune checkpoint blockade has demonstrated therapeutic potential in TNBC, clinical responses remain inconsistent. Initially, the lack of full-length ERα-66 suggested that estrogen signaling was not linked to the development and progression of ER- breast cancers. However, subsequent studies have shown that subsets of ER- cells retain rapid non-genomic ER signaling pathways by expressing the ERα-36 variant, which likely contributes to TNBC development and progression. Therefore, this receptor could be considered a potential target for anticancer drugs [24]. This suggestion was partially confirmed by a study showing that naturally occurring pterostilbene inhibits ERα-36 and induces apoptosis in MCF7/ER36 breast cancer cells [40].

A promising therapeutic strategy may also target epigenetic alterations typically associated with TNBC pathogenesis and treatment resistance, such as aberrant DNA methylation, promoter hypermethylation of tumor suppressor genes, and dysregulated histone-modifying enzymes, including EZH2 and histone deacetylases (HDACs). As a result, epigenetic alterations in TNBC significantly affect tumor proliferation, survival, and metastasis. Therefore, to enhance therapeutic effectiveness in TNBC, combining immunotherapy with epigenetic inhibitors might be a promising option [41].

It is now clear that TNBC, initially considered a single disease, presents a spectrum of biologically diverse tumors, characterized by distinct molecular and clinical courses [42]. Emerging multi-omics approaches further deepened insights into TNBC and may pave the way to new treatment options.

### 2.4. ER Modulators—Overcoming Resistance

Breast cancer is a heterogeneous disease and often becomes resistant to treatment. Such resistance may be primary, resulting from intrinsic tumor insensitivity, or acquired, developing through the selective expansion of resistant cell populations during therapy. Multiple mechanisms, including enhanced DNA repair, antiapoptotic signaling, activation of autophagy, increased drug efflux, and genetic alterations in TP53, PIK3CA, and BRCA1/2, drive resistance. TME, particularly cancer-associated fibroblasts and VEGF-mediated angiogenesis, further contributes to therapeutic failure [37,43,44,45]. Both intrinsic and acquired resistance limit the long-term efficacy of endocrine therapies, especially in TNBC and advanced stages of the disease [46,47]. Endocrine resistance has been explained by several mechanisms. A common type of resistance involves the loss or mutation of ERα, reducing responsiveness to hormonal therapies like tamoxifen. Mutations in the ERα gene occur in less than 5% of primary tumors, whereas they are significantly increased to up to 50% in metastatic resistance tumors [48]. Many mechanisms contribute to resistance to ER modulators. Evidence suggests that the non-canonical ER signaling pathway could be associated with resistance to endocrine therapy and poor prognosis in breast cancer [49,50], especially given that the non-genomic pathway is favored over the genomic pathway during breast cancer progression [51]. Recent studies on breast cancer cells have shown that mutations in ERα can trigger epithelial–mesenchymal transition and enhance proliferation and invasion of the cancer cells [52]. Hence, the search for alternative ER modulators is very necessary and desirable. One option could be a GPER inhibitor, which has induced apoptosis and reduced TNBC size in mice [53].

It should also be stressed that recently, the search for alternative treatment options for resistant breast cancer has been increasingly focused on the heterogeneous nature of the disease. Understanding the molecular factors that influence the high variability in individual breast cancer cases may allow for optimized treatment to circumvent recurrent resistance and minimize side effects. Typical breast cancer classification omits the diversity of cases within the same molecular profile subtype of the disease. Multiple heterogeneity factors should be taken into account, starting with intertumor heterogeneity arising from genomic, transgenomic, and phenotypic differences among the patients and finishing with intratumor heterogeneity driven by clonal diversity, epigenetic plasticity, and interactions with the TME [2,42].

## 3. Major/Key Enzymes Involved in Estrogen Synthesis and Their Modulators

### 3.1. Aromatase (CYP19A1) in Breast Cancer and Its Inhibitors

#### 3.1.1. Characteristics, Function, and Breast Cancer Clinical Relevance

Aromatase (EC 1.14.14.14) belongs to the cytochrome P450 (CYP) superfamily and is classified as CYP19A1. Aromatase is a 57 kDa integral membrane protein of the endoplasmic reticulum, anchored to the membrane by its N-terminal transmembrane domain. It catalyzes the transformation of C19 androgens, including testosterone and androstenedione, into C18 estrogens, such as E1 and E2 [54]. Aromatase activity is highly specific because it is the sole enzyme in humans capable of converting androgens to estrogens [55,56]. Aromatization catalyzed by aromatase occurs primarily in the endoplasmic reticulum of estrogen-producing cells and is important for maintaining normal estrogen levels, especially in postmenopausal women, whose estrogen production is significantly reduced [57]. The conversion of androgens to estrogens occurs in three steps and requires NADPH-cytochrome P450 reductase, which supplies the electrons for the hydroxylation reactions [58]. Aromatase is expressed in various tissues, including the ovaries, testis, adipose tissue, breast, adrenal gland, and skin, and to a lesser extent in other tissues [57,59]. Importantly, aromatase is commonly expressed in breast cancer tissue, leading to high local estrogen production [60].

#### 3.1.2. Aromatase Inhibitors

Taking into account the crucial role of aromatase in estrogen formation, the possibility of its inhibition for therapeutic purposes was the subject of intensive studies, leading to the development of commercial drugs used for breast cancer treatment, particularly in postmenopausal women. Based on their development history and characteristics, reflecting advancements in specificity and efficacy over time, three generations of aromatase inhibitors were described [54]. The first generation included nonselective agents, such as aminoglutethimide and testolactone. These compounds inhibit multiple enzymes involved in steroid synthesis, often leading to side effects, such as adrenal insufficiency [59,61]. The second generation of aromatase inhibitors exhibited improved selectivity. The first selective steroidal inhibitor of this group used clinically was formestane, while fadrozole represented a non-steroidal option with enhanced selectivity [62]. Because of low potency, lack of specificity, or unfavorable side effects, studies on these compounds were discontinued. Aromatase inhibitors currently used in the clinic are referred to as third-generation inhibitors. According to their structures and mechanisms of action, they are similarly divided into steroidal and non-steroidal inhibitors/blockers, such as second-generation inhibitors.

The representative of the first group is exemestane, which is derived from androstane-3,17-dione, the natural substrate of aromatase, and is an irreversible inhibitor that covalently binds to the enzyme. Non-steroidal aromatase inhibitors, anastrozole and letrozole, are imidazole derivatives that interact with the heme moiety of this CYP enzyme and act as reversible competitive inhibitors [59]. These commercially available compounds, which have high specificity for aromatase, do not interfere with the biosynthesis of other steroid hormones and are, to date, the first-line breast cancer therapy for postmenopausal women [58].

Although these compounds are effective breast cancer drugs, resistance has been observed in numerous cases, particularly in recent years. Thus, the search for new aromatase inhibitors remains necessary. A new approach involves creating molecules that can simultaneously inhibit two or more enzymes, including those involved in estrogen synthesis or signaling, such as combining SERMs with aromatase inhibitors [58], as well as other pathways. An example of this approach is the combination of aromatase and COX2 inhibitors, which reduce both estrogen production and inflammation and induce apoptosis in cancer cells [63]. Recently, an important observation was made regarding resistance to aromatase inhibitors. Müller et al. [64] found that Glycine-N-Acyltransferase Like 1 (GLYATL1) expression is upregulated in aromatase inhibitor-resistant breast cancer cell models and in patients undergoing therapy with these drugs, and that this correlates with poorer survival. Moreover, they demonstrated that GLYATL1 promotes resistance to estrogen deprivation by elevating succinate levels and altering epigenetic histone marks associated with active transcription. Therefore, GLYATL1 could be a potential target to overcome aromatase inhibitor resistance in ER+ breast cancer, as it acts as a metabolic and epigenetic mediator of resistance to therapy.

Another interesting approach to overcome resistance to aromatase inhibitors is to develop allosteric inhibitors of this enzyme. The molecules with such activity, in contrast to competitive inhibitors binding to the catalytic center of the enzyme, could achieve the maximal inhibitory effect without complete inhibition of estrogen production, which leads to undesired side effects of therapy. So far, however, compounds with such activities have been tested only in silico. Thus, the location of multiple ligand-binding sites on aromatase is only predicted [65].

### 3.2. Steroid Sulfatases in Breast Cancer and Their Possible Modulations

#### 3.2.1. Characteristics, Function, and Breast Cancer Risk/Clinical Relevance

Steroid sulfatase (EC 3.1.6.2, STS), also called aryl sulfatase C, belongs to the mammalian sulfatase family and plays a crucial role in steroid production. It breaks down inactive steroid sulfates such as estrone-3-sulfate (E1S) and dehydroepiandrosterone-3-sulfate (DHEAS), which are precursors to the formation of active estrogens and androgens [66]. STS is a transmembrane protein predominantly associated with the endoplasmic reticulum, with smaller fractions found in the Golgi apparatus and at the cell surface [67]. The existence of a nuclear STS isozyme was also confirmed [68]. The tissue distribution of STS varies considerably among mammals. The placenta is the most abundant source of STS in humans. However, enzyme activity has also been found in numerous other tissues, including the adrenal glands, ovary, testis, prostate, skin, and brain. STS was also detected in malignant prostate and breast tissues, as well as in cancer cell lines (e.g., LNCaP, DU-145, PC-3, MCF7). It was reported that STS expression in some malignant tissues (breast, prostate) is significantly higher than in normal tissues, suggesting a potential role of STS in hormone-dependent cancers, including breast cancer. Thus, considering that sulfated steroids are neither ligands of steroid receptors nor active hormones, the inhibition of STS could be a desirable strategy to inactivate the biological functions of steroids and could serve as a druggable target for breast cancer treatment [69]. According to in vitro studies, STS has several hundred times higher activity in breast tissue than aromatase [70,71]. Moreover, high mRNA levels of STS in ER+ breast tumors were an independent prognostic indicator of relapse-free survival, with higher expression associated with a poor prognosis [72].

#### 3.2.2. STS Inhibitors

Given the role of STS in breast and other hormone-dependent cancers, effective STS inhibitors have been sought after in the last few decades. The earliest STS inhibitors were synthetic and naturally occurring steroid-related compounds, including the most potent in this group, 5-androstene-3β,17β-diol-3-sulfate. However, these alternative STS substrates could be metabolized in vivo to form estrogenic forms with ER-binding affinity. Non-steroidal sulfamates were among the first non-steroidal STS inhibitors discovered. EMATE (estrone-3-O-sulfamate), although promising in vitro, did not advance to clinical trials due to serious side effects. Coumarin sulfamates, such as 7-O-sulfamate (COUMATE), inhibited STS activity in MCF7 breast cancer cells by >90% at 10 µM; therefore, several derivatives from this group were evaluated. Among them, Irosustat (STX64) was the subject of phase I clinical trials, which showed that inhibition of STS may be beneficial for patients in the early stages of breast cancer. Interestingly, STS inhibition also reduced aromatase and HSD17B1 expression in these patients [73].

So far, no STS inhibitors have been market-ready. Consequently, efforts focus on identifying new candidates with enhanced features and on creating multi-target drugs capable of modulating multiple enzymes in the steroidogenic pathway [74]. The strategy of combining STS inhibitors with aromatase inhibitors has already proven successful in treating ER+ breast cancer patients [73].

### 3.3. 17β-Hydroxysteroid Dehydrogenases in Breast Cancer and Their Possible Modulations

#### 3.3.1. Characteristics, Function, and Breast Cancer Risk/Clinical Relevance

The 17β-hydroxysteroid dehydrogenase enzymes (HSD17Bs, EC1.1.1.62) catalyze NAD(P)(H)-dependent oxidoreduction of hydroxyl and keto groups at position C17 of androgens and estrogens, thereby regulating the intracellular availability of steroid hormone ligands to their nuclear receptors. So far, fourteen mammalian HSD17Bs have been identified. Except for HSD17B5, which belongs to the aldo-keto reductase (AKR) family, are all part of the short-chain dehydrogenase/reductase (SDR) family [3]. Human HSD17Bs differ in nucleotide cofactor requirements, subcellular localization, and expression patterns. Consequently, they are generally divided into oxidative enzymes (HSD17B types 2, 4, 6, 8, 9, 10, 11, 14), which catalyze NAD+-dependent inactivation, and reductive enzymes (HSD17B types 1, 3, 5, 7), which are NADPH-dependent and lead to the formation of active receptor ligands. Therefore, HSD17Bs were initially identified by their ability to interconvert active and inactive forms of sex steroids. It is now well known that the substrates of these enzymes also include fatty acids, bile acids, and retinoids. Functional divergence is often reflected in their subcellular localization [75].

Among the various HSD17B types, HSD17B1, particularly the HSD17B1:HSB17B2 redox balance, plays the most important role in hormone-dependent diseases, including breast cancer [59]. HSD17B1, localized in the cytosol, acts as a 17-hydroxysteroid reductase and converts E1 into the most potent estrogen, E2. In contrast, HSD17B2, located in the endoplasmic reticulum, catalyzes the reverse reaction, i.e., the inactivation of E2 by oxidizing it to E1 [76]. These enzymes control the final step in the local formation of E2 and are expressed in a variety of tissues. In addition to female and male reproductive tissues, they are expressed in the bone, lungs, gastrointestinal tract, and central nervous system [77,78], confirming their enzymatic activity on substrates other than E1 and E2.

In breast cancer and other estrogen-dependent diseases, the interplay between these two enzymes is disrupted, leading to an imbalance between estrogen activation and inactivation. The crucial role of high HSD17B1 in these diseases is indicated by the correlation of its high expression with a high E2/E1 ratio [79]. Several studies have demonstrated that inhibition of HSD17B1 prevents the proliferation of breast cancer cells in vitro and reduces tumor volume and E2 plasma concentration in in vivo models. Moreover, it was shown that, in addition to its role in E2-dependent gene transcription, HSD17B1 modulates gene expression via mechanisms independent of steroid actions. Those mechanisms may include ligand-independent gene transcription of estrogen receptor alpha (ERα), whose expression is positively correlated with that of the enzyme and may implicate the HSD17B1’s role in anti-apoptotic pathways [80].

HSD17B2, which antagonizes HSD17B1 activity, was found to be downregulated in malignant breast cancer compared to benign tumors [81], and its gene expression showed an inverse correlation with E2 levels [82]. This HSD17B variant plays a key role in hormonal balance in postmenopausal women, when E2 levels decline [1]. Higher expression of HSD17B2 was observed in ERα- breast cancer [83].

Although HSD17B1 and HSD17B2 play the most important roles in breast cancer pathogenesis, other members of this enzyme family may also contribute to hormonal imbalance in this disease. In this regard, HSD17B4, localized in peroxisomes and overexpressed in breast cancer, similarly to HSD17B2, mediates the transformation of E1 to E2; however, its activity is much lower than that of HSD17B2 [84].

Cytosolic HSD17B5 expression is significantly higher in breast tumor specimens compared with normal tissues and has been linked to a worse prognosis [85]. Analysis of the proteome in MCF7 cells showed that HSD17B5 knockdown upregulated proteins that can enhance breast cancer development. One of them is GRP78, an inhibitor of apoptosis. Therefore, it was concluded that HSD17B5 may not be a potent target for breast cancer treatment, but its low level of expression could serve as a poor prognostic factor [86].

HSD17B6, localized in the endoplasmic reticulum, primarily mediates the conversion of dihydrotestosterone (DHT) to 3α- and 3β-diol [87]. In TNBC, HSD17B6 expression has been linked to better outcomes, likely by enhancing ERβ signaling [88]. HSD17B7 is also localized in the endoplasmic reticulum and is ubiquitously expressed, catalyzing the conversion of E1 to E2 and the reduction of DHT. Knocking out the HSD17B7 gene in breast cancer cell lines led to reduced proliferation [89]. This enzyme is primarily involved in cholesterol synthesis, but its dual role in producing E2 and degrading DHT makes it a potential therapeutic target for treating estrogen-dependent cancers [90].

Cytosolic HSD17B14, expressed in several tissues besides sex organs and using NAD+ as a cofactor, catalyzes the oxidation of E2 to E1 and androstenediol to DHEA [3]. However, its activity in this context appears to be lower than that of HSD17B2 [91]. Higher expression in breast cancer has been observed [92]. Moreover, Qureshi et al. [93] demonstrated that HSD17B14 overexpression decreased intracellular E2 and increased E1, along with increased expression and secretion of pro-inflammatory cytokines in HSD17B14-transduced ER+ breast cancer cell lines. This observation might explain the increased risk of ER+ breast cancer in obese postmenopausal women and has paved the way for prevention and therapy.

Disrupted expression and activity of certain HSD17B types in breast cancer, as described above, make them potential drug targets and prognostic markers. In this regard, the study by Gunnarsson et al. [94,95] in two postmenopausal cohorts demonstrated that a high HSD17B1-to-HSD17B2 ratio, as well as high HSD17B1 alone, was associated with a worse prognosis and an increased risk of recurrence in patients with ERα-positive tumors. Moreover, high tumoral HSD17B2 expression or a high HSD17B2-to-HSD17B1 ratio was associated with improved prognosis and a reduced risk of recurrence in patients with ERα(+) tumors. Additionally, higher HSD17B1 expression was associated with an increased risk of recurrence at 5 years [94,95]. When the copy number variation of the HSD17B1 gene was analyzed, increased copy number was correlated with decreased breast cancer survival [96]. Interestingly, the HSD17B1-to-HSD17B2 ratio is a good indicator of the benefits of tamoxifen treatment [97]. A recent study of premenopausal women diagnosed with breast cancer in the Danish population confirmed that HSD17B1 may be a prognostic marker for recurrence and that HSD17B2 may be predictive of response to tamoxifen therapy [98].

Some studies have reported that HSD17B14 may be as useful as an indicator of tamoxifen treatment effect on recurrence-free survival in ERα+ lymph node-negative breast cancer patients [91].

#### 3.3.2. HSD17Bs Modulators

Based on the information provided in the previous section, HSD17Bs may be considered attractive targets for treating breast cancer and other hormone-dependent diseases. However, the complexity of these enzymes’ activity, often linked to their action on specific receptors, requires the development of inhibitors that lack agonist activity at the receptor involved in the signaling pathway. Moreover, the opposing actions of certain HSD17B types, such as HSD17B1 and HSD17B2, require inhibitors selective for the critical type (e.g., HSD17B1) without inhibiting the other (e.g., HSD17B2). Therefore, inhibitors of HSD17B1 should not also inhibit HSD17B2. Since both E1 and E2 primarily act via nuclear ERα and ERβ, and because E1 has weaker estrogenic activity due to its lower ER affinity than E2, any potential modulators of HSD17B1 and HSD17B2 should lack agonistic activity at ER. Over the last 50 years, particularly in the 21st century, numerous HSD17B inhibitors have been developed. However, most of them were tested only in vitro and in animal models [59]. Several comprehensive reviews on this subject were published recently, including one by Donald Poirer, a leader in this field [5]. Therefore, here we mention only the basic strategies in searching for HSD17B inhibitors/modulators.

Due to its role in E2 synthesis and estrogen-dependent breast cancers, HSD17B type 1 has been one of the most thoroughly studied potential therapeutic targets. Inhibitors of HSD17B1 are generally classified into two groups: steroidal (derivatives of E1 or E2) and non-steroidal, where molecules mimic the steroid scaffold. For the first group, a series of compounds based on the estrone backbone with C15 modifications was initially developed. According to 3D crystal structure studies, after the substrate docks in the catalytic site, C15 becomes exposed outside the enzyme–substrate complex, making it accessible for modifications aimed at enhancing chemical and pharmacological properties. In addition, C15 is engaged in the ER–ligand complex, suggesting that modification at this carbon would prevent any unwanted estrogenic action. The therapeutic efficacy of this compound was thoroughly tested in various in vivo models. Messinger et al. induced breast cancer in mice through subcutaneous injection of MCF7 cells and observed a reduction of over 80% in tumor growth [99]. The same compound or its derivatives demonstrated effectiveness in orthotopic breast cancer models [100]. More recently, a series of estradiol derivatives with C16 beta-m-carbamoylbenzyl modifications was developed [101]. CC-156, the first in this series, showed some estrogenic activity, which was eliminated upon C3-bromoethyl modification, leading to compound PBRM and subsequent analogs. PBRM, an irreversible inhibitor of HSD17B1, showed therapeutic effectiveness in a breast cancer mouse model [1]. Tumor xenografts were induced subcutaneously using the T47D breast cancer cell line [1,101]. Among non-steroidal inhibitors, HSDB171 hydroxyphenylnaphthols were described as potent and selective inhibitors of this enzyme [102]. Other inhibitors in this group include: bis(hydroxyphenyl) substituted arenes, bicyclic substituted hydroxyphenyl methanones, hydroxybenzothiazole and hydroxybenzothiophene derivatives, thiophenepyrimidinone, biphenyl ketones, biphenylols, and imidazole [59].

In addition to synthetic compounds, inhibitors of HSD17Bs are sought among derivatives of phytochemicals such as flavonoids, cinnamic acid, and coumarins. Niinivehmas et al. [103] reported a series of 3-phenylcoumarin analogs as inhibitors of recombinant human HSD17B1. The molecular basis of inhibition was elucidated through a docking-based SAR analysis, and inhibition of HSD17B2, as well as cross-reactivity against ERα, aromatase, CYP1A2, and monoamine oxidases, was evaluated. The results showed that the coumarin core can be engineered to block E2 synthesis via either the sulfatase or aromatase pathway by adding a 3-phenyl or 3-imidazole ring, respectively.

As mentioned previously, the most important requirement for HSD17B1-specific inhibitors is that they should lack agonist activity on ER and not inhibit the oxidative forms of HSD17B, particularly HSD17B2 and HSD17B14. Therefore, although a large number of potential HSD17B inhibitors have been developed, only one reached the stage of clinical trial, and not in the context of breast cancer, but in endometriosis [59]. An alternative method to inhibit HSD17B1 that avoids issues with estrogenic activity or off-target effects is to use siRNA to downregulate the enzyme. Such an elegant approach was recently tested in a mouse model of breast cancer [104].

Considerable work has been done to develop both steroidal and non-steroidal inhibitors of HSD17B2 [5,79]. However, using an inhibitor of HSD17B2 would be the strategy to maintain adequate levels of E2 in postmenopausal women to avoid osteoporosis. In premenopausal women, rather than activators of these enzymes, inhibitors would be desirable to reduce breast cancer risk. To our knowledge, no attempts have been made in this direction so far. The same observation might apply to efforts to develop inhibitors of HSD17B14, as this enzyme catalyzes the oxidation of 17β-hydroxysteroids to 17-ketosteroids and, like HSD17B2, deactivates E2.

HSD17B5 is a member of the aldo-keto reductase (AKR) superfamily, unlike other HSD17Bs, which are members of the SDR. It is involved in the production of aromatase substrates and ultimately in the formation of estrogens, including E2, making it an interesting therapeutic target for treating hormone-dependent cancers. Several steroid-based inhibitors based on C3- and C17-substituted estrogens have been developed. Interestingly, non-steroidal inhibitors, including repurposed NSAIDs, have also been claimed in multiple patents. However, most of these enzyme inhibitors require optimization before testing in animal models and humans [105].

HSD17B7, as mentioned in the previous section, is a multifunctional enzyme thought to be involved in the synthesis of cholesterol and the estrogenic steroid hormone E2, as well as in the degradation of the androgenic hormone DHT. Therefore, steroid derivatives were tested as inhibitors to identify molecules selective for one or the other enzymatic transformation. This approach was used by Lin’s group [106]. Their investigation identified 4-aza-androstane, a known inhibitor of HSD17B7, as selective for the reduction of E1 to E2.

To sum up, many inhibitors and modulators of HSD17Bs, which are critically involved in the redox balance of estrogens and thus in breast cancer pathogenesis, have been discovered, and basic strategies have been developed. However, inhibitors with the required characteristics, i.e., lacking agonistic effects on ER and selective for a single HSD17B type, are still sought. One option is to apply a biotechnological approach, such as siRNA.

**Figure 1:**
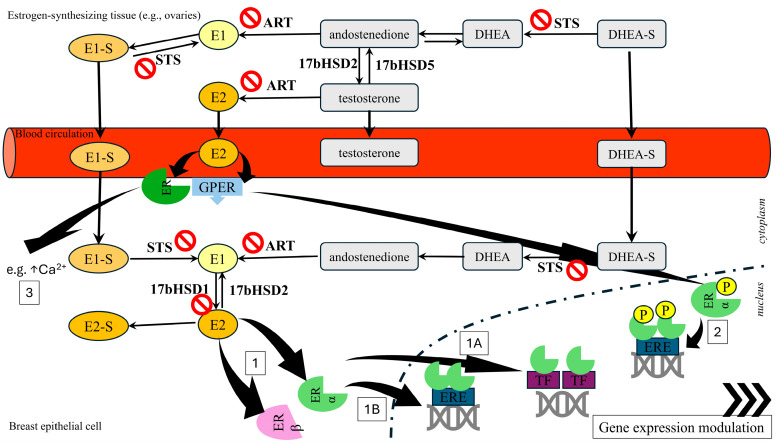
Steroid hormones can be synthesized by secreting dehydroepiandrosterone sulfate (DHEA-S) or estrone sulphate (E1-S), which are then locally metabolized into active hormones. Therefore, in postmenopausal women, sex steroids, including estrogens, are predominantly produced in extragonadal tissues from adrenal precursors, a system termed “intracrinology”. Three enzymes are particularly important in estrogen synthesis, both in gonadal and intracrine contexts: aromatase (CYP19), steroid sulfatase (STS), and 17β-hydroxysteroid dehydrogenases (17bHSD). Locally generated active estrogens can bind to estrogen receptors and transduce genomic (1, 2) estrogen receptor (ER) signaling (1A—indirect binding to DNA via transcription factors, TF; 2—ligand-independent binding to the DNA of membrane ERs activated by kinase signaling phosphorylation; 1B—direct translocation to the nuclei, binding to the consensus response element, ERE, in the DNA). Simultaneously, the circulating free 17β-estradiol (E2) can activate transmembrane G protein-coupled estrogen receptors (GPER) on the surface of breast tissue and transduce non-genomic (3) ER signaling, resulting in, e.g., an increase in intracellular calcium level. Other abbreviations: ART—aromatase, 17bHSD—17β-hydroxysteroid dehydrogenase, STS—steroid sulfatase.

The above-described roles of estrogens, ER, and enzymes involved in metabolism, as well as the mechanism of estrogen action in mammary epithelial cell function, are summarized in Figure 1.

Overall, the major estrogen-synthesis enzymes, along with ER modulators, are certainly attractive therapeutic targets (Table 1). However, the mechanisms of their inhibitors’ activity and possible side effects still require more extensive preclinical and clinical studies in most cases. Among the three described enzymes, the most promising seems to be HSD17B, as they control the final step in the local formation of E2. On the other hand, developing single molecules interfering with different steps of E2 synthesis and/or ER could reduce the side effects of selective compounds. Such hybrid molecules are synthesized, but their activities so far have been confirmed only in vitro studies.

## 4. Conclusions and Perspectives

**Figure 2:**
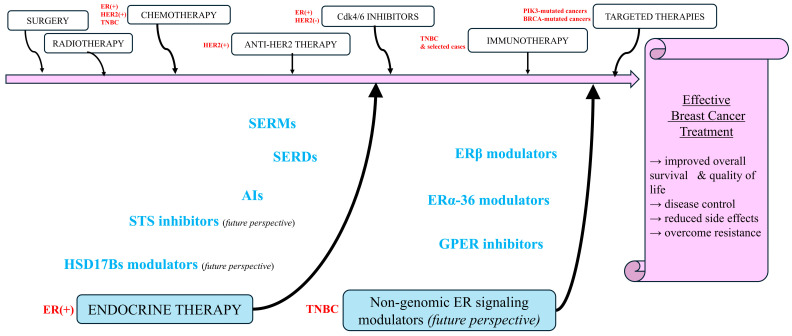
An integrated scheme summarizing current therapies and therapies under investigation with particular emphasis on future perspectives of modulating estrogen receptors and enzymes involved in estrogen synthesis in the mammary gland tissue (ER—estrogen receptor, HER—human epidermal growth factor, TNBC—triple-negative breast cancer, BRCA—breast cancer gene, PIK3—phosphatidylinositol 3-kinase, SERMs—selective estrogen receptor modulators, SERDs—selective estrogen receptor degraders, AI—aromatase inhibitors, STS—steroid sulfatase, HSD17Bs—17β-hydroxysteroid dehydrogenases, GPER—transmembrane G protein-coupled estrogen receptor).

Targeting estrogen receptors, particularly when combined with aromatase inhibitors, is an effective strategy for treating ER+ breast cancers in most cases. However, searching for novel targets involved in the initiation and development of this disease remains a priority (Figure 2). One reason is that complete depletion of estrogens reduces bone density and increases the risk of cardiovascular problems. Other reasons include unfavorable side effects and the development of resistance to commercial drugs.

Among the new targets, HSD17B enzymes are the most interesting as they control the final step of E2 synthesis. Recent developments that have enabled the generation of new classes of inhibitors, particularly for HSD17B1, may pave the way for some to advance through preclinical and clinical studies and ultimately become the first drugs targeting HSD17B enzymes. An interesting alternative to searching for chemical inhibitors might be a biotechnological approach, such as suppressing HSD17B1 with siRNA.

Exploitation of multitarget strategies is another promising area of study aimed at improving breast cancer treatment. In this regard, recent studies suggest the possibility of developing aromatase/SERM dual-target agents that could be more effective than single-target agents targeting the ER or a single enzyme. Similarly, combining STS inhibitors with aromatase inhibitors has already been shown to be a successful approach in ER+ breast cancer patients. The recently proposed application of machine learning [107] to optimize synergistic drug combinations might be helpful in the further development of multitarget strategies for breast cancer treatment. However, this concept has not yet been confirmed in profound preclinical in vivo studies and consequently in clinical trials.

Overcoming resistance to currently used drugs is another problem to be solved. Repurposing a COX-2 inhibitor as an anti-cancer agent may overcome aromatase resistance. Equally important is a better understanding of the mechanism of resistance. In this regard, GLYATL1, a metabolic and epigenetic mediator of endocrine therapy resistance, may be a potential target to overcome resistance to aromatase inhibitors and other inhibitors targeting estrogen-synthesizing enzymes in ER+ breast cancer. In contrast to ER+, TNBC has limited treatment options due to the absence of ERα, rendering endocrine therapy ineffective. A recent discovery that epigenetic drugs, such as DNA methyltransferase and histone deacetylase inhibitors, can restore the expression of ERα may increase treatment options for this type of breast cancer. Finally, it has been stressed that besides the therapeutic targets, cancer cell heterogeneity and TME are the important components that determine the drug response [108]. Therefore, future work should also aim to uncover how tumor heterogeneity affects TME remodeling, as well as how the latter affects drug responses.

## Figures and Tables

**Table 1:** The summary of estrogen receptor modulators and enzyme synthesis inhibitors described in the text.

Molecular Target	Modulator/Inhibitor	References
	raloxifene	[34]
	ospemifene	[34]
	lasofoxifene	[34]
ER	bazedoxifene	[34]
	fulvestrant	[35]
	elacestrant	[36]
	camizestrant	[36]
	giredestrant	[36]
	imlunestrant	[36]
	amcenestrant	[36]
	aminoglutethimide	[6,59]
	testolactone	[6,59]
	formestane	[62]
	fadrozole	[62]
CYP19A1	exemestane	[59]
	anastrozole	[59]
	letrozole	[59]
	imidazole	[59]
	GLYATL1	[64]
STS	COUMATE	[73]
	STX64	[73]
	CC-156	[1]
HSD17B	PBRM	[1]
	hydroxyphenylnaphthols	[59]
	3-phenylcoumarin	[103]

## Data Availability

No new data were created or analyzed in this study. Data sharing is not applicable to this article.

## Abbreviations

The following abbreviations are used in this manuscript:

CERANs	Complete estrogen receptor antagonists
COUMATE	Coumarin 7-O-sulfamate
CYP	Cytochrome P450
CYP19	Aromatase
DHEA	Dehydroepiandrosterone
DHEA-S	Dehydroepiandrosterone sulfate
E1	Estrone
E2	17β-estradiol
EMATE	Estrone-3-O-sulfamate
ER	Estrone receptor
EREs	Estrogen response elements
GLYATL1	Glycine-N-Acyltransferase Like 1
GPER	Transmembrane G protein-coupled estrogen receptor
HDACs	Histone deacetylases
HSD17B	17β-hydroxysteroid dehydrogenases
HER2	Human epidermal growth factor receptor 2
PR	Progesterone receptor
PROTACs	Proteolysis-targeting chimeras
SERCAs	Selective estrogen receptor covalent antagonists
SERDs	Selective estrogen receptor degraders
SERMs	Selective estrogen receptor modulators
STS	Steroid sulfatase
TME	Tumor microenvironment
TNBC	Triple-negative breast cancer
